# Comparing surgical outcomes of approaches to adrenalectomy — a systematic review and network meta-analysis of randomised clinical trials

**DOI:** 10.1007/s00423-023-02911-7

**Published:** 2023-05-05

**Authors:** Matthew G. Davey, Éanna J. Ryan, Noel E. Donlon, Odhrán K. Ryan, Mohammed Al Azzawi, Michael R. Boland, Michael J. Kerin, Aoife J. Lowery

**Affiliations:** 1 Discipline of Surgery, The Lambe Institute for Translational Research, National University of Ireland, Galway, Galway, H91YR71 Ireland; 2 Department of Surgery, Galway University Hospitals, Galway, H91YR71 Republic of Ireland; 3 Royal College of Surgeons in Ireland, 123 St. Stephens Green, Dublin 2, D02YN77 Ireland; 4 Surgical Professorial Unit, St. Vincent’s University Hospital, Elm Park, Dublin 4, D04 T6F4 Ireland

**Keywords:** Adrenal surgery, Adrenalectomy, Surgical oncology, Oncological outcomes

## Abstract

**Background:**

No randomised clinical trials (RCTs) have simultaneously compared the safety of open (OA), transperitoneal laparoscopic (TLA), posterior retroperitoneal (PRA), and robotic adrenalectomy (RA) for resecting adrenal tumours.

**Aim:**

To evaluate outcomes for OA, TLA, PRA, and RA from RCTs.

**Methods:**

A NMA was performed according to PRISMA-NMA guidelines. Analysis was performed using R packages and Shiny.

**Results:**

Eight RCTs with 488 patients were included (mean age: 48.9 years). Overall, 44.5% of patients underwent TLA (217/488), 37.3% underwent PRA (182/488), 16.4% underwent RA (80/488), and just 1.8% patients underwent OA (9/488). The mean tumour size was 35 mm in largest diameter with mean sizes of 44.3 mm for RA, 40.9 mm for OA, 35.5 mm for TLA, and 34.4 mm for PRA (*P* < 0.001). TLA had the lowest blood loss (mean: 50.6 ml), complication rates (12.4%, 14/113), and conversion to open rates (1.3%, 2/157), while PRA had the shortest intra-operative duration (mean: 94 min), length of hospital stay (mean: 3.7 days), lowest visual analogue scale pain scores post-operatively (mean: 3.7), and was most cost-effective (mean: 1728 euros per case). At NMA, there was a significant increase in blood loss for OA (mean difference (MD): 117.00 ml (95% confidence interval (CI): 1.41–230.00)) with similar blood loss observed for PRA (MD: − 10.50 (95% CI: − 83.40–65.90)) compared to TLA.

**Conclusion:**

LTA and PRA are important contemporary options in achieving favourable outcomes following adrenalectomy. The next generation of RCTs may be more insightful for comparison surgical outcomes following RA, as this approach is likely to play a future role in minimally invasive adrenalectomy.

**PROSPERO registration:**

CRD42022301005.

**Supplementary Information:**

The online version contains supplementary material available at 10.1007/s00423-023-02911-7.

## Introduction

While tumours of the adrenal glands were traditionally resected through open adrenalectomy (OA) [1], the contemporary approach to adrenal lesions has evolved to minimally invasive techniques (both transperitoneal laparoscopic and more recently retroperitoneoscopic), replacing traditional open resections [2, 3]. In recent times, the surgical approach has further evolved with the adoption of robotic technology as the next iteration of minimally invasive surgery (MIS). MIS was originally pioneered in colorectal surgery and the evolution of its use has extended to endocrine surgery, such that minimally invasive adrenalectomy is now considered the gold standard for resecting most lesions of the adrenal gland [4–8]. Laparoscopic adrenalectomy was first described by Gagner et al. [9] and Higashihara et al. [10] in 1992, which involved a transperitoneal laparoscopic approach to adrenalectomy (TLA), before Walz et al. described a posterior retroperitoneal approach to resecting the adrenal gland (PRA) [11]. While the European Society of Endocrine Surgeons (ESES) continues to recommend OA for the resection of adrenocortical carcinoma (ACC) and cancers of 8 cm or greater in size [12], performing laparoscopic adrenalectomy is considered advantageous over OA if possible, due to a reported reduction in post-operative pain, major morbidity, length of hospital stay (LOS), surgical site infection, and faster recovery time [13]. Notwithstanding, performing laparoscopic adrenalectomy does confer unavoidable limitations: the operating surgeon has limited instrument movement/dexterity compared to OA and the natural physiological hand tremor becomes amplified at laparoscopy. Furthermore, the two-dimensional projection of images through the camera limits the view of the surgical field in its entirety [14, 15]. In addition, laparoscopic approaches become challenging in complex patients, including those with large tumours, with phaeochromocytomas, and in those with increased body habitus. In such complex cases, conversion from laparoscopic to OA typically occurs in approximately 3–5% of cases [16, 17]. As described, the ESES guidelines further reflect the importance of OA in specific circumstances, as this approach remains the gold standard for resections of ACC and large tumours (8 cm in size or greater) [12].

Robotic surgery first made its debut as a technique for performing adrenalectomy in 2002 [18]. Robotic technology is an attractive addition to the surgeon’s armamentarium with theoretical advantages over conventional laparoscopic techniques such as improved dexterity (including more flexible and intuitive instrument control), a wristed instrument to eliminate physiological tremor, and a three-dimensional visual field which is non-inferior to laparoscopy [19, 20]. Shortcomings of robotic adrenalectomy (RA) are the longer operative time, increased expenses associated with purchasing and maintaining equipment and training operators, a steep learning curve, and poorer cost-effectiveness in low-volume centres [21–24].

Previous meta-analyses have addressed the oncological and surgical safety of RA compared to laparoscopic adrenalectomy [25–27], while others have evaluated the utility of both TLA and PRA over OA in achieving safe and effective resection of lesions of the adrenal glands [13, 28–30]. While these meta-analyses compare the outcomes for two approaches to adrenalectomy using studies of retrospective design, there are no previous meta-analyses performed evaluating the safety of RCTs comparing the safety of all four approaches to adrenalectomy. Therefore, applying network meta-analysis (NMA) methodology is advantageous if employed here as it facilitates simultaneous comparison of OA, TLA, PRA, and RA [31, 32]. Accordingly, the aim of the current systematic review and network meta-analysis of randomised clinical trials (RCTs) was to evaluate the post-operative surgical and oncological outcomes of OA, TLA, PRA, and RA.

## Methods

A systematic review was performed in accordance with the ‘Preferred Reporting Items for Systematic Reviews and Meta-Analyses’ (or PRISMA) extension statement for reporting of systematic reviews incorporating network meta-analyses of healthcare interventions [33]. Local institutional ethical approval was not required as all data used in this analysis was obtained from a previously published resource. This study was prospectively registered with the International Prospective Register of Systematic Reviews (PROSPERO): CRD42022301005.

### Study eligibility

For inclusion in this analysis, studies had to meet the following inclusion criteria: (1) prospective randomised trials (i.e. RCTs) which compared any peri-operative implications and complication rates following different approaches to adrenalectomy (i.e. any post-operative complications; intra-operative blood loss; duration of the operation; conversion to open adrenalectomy; post-operative pain at 12 h post-operatively (D0), 24 h post-operatively (D1), and 48 h post-operatively (D2); cost of procedure; and length of stay (LOS)), irrespective of clinical, radiological, or oncological indication for surgery, (2) studies had to provide full-text manuscripts, and (3) be published in the past 20 years (2001–2021). Included studies were expected to report patient demographics and to report on the primary outcomes of interest. Studies failing to meet these strict eligibility criteria were not considered for inclusion. Conference abstracts and studies not published in the English language were excluded.

### Population, Intervention, Comparison, Outcomes (PICO)

Using the PICO framework [34], the aspects the authors wished to address were:

- Population — Patients aged 18 years or older who were due to undergo adrenalectomy for any indication.
- Intervention — Any patient eligible for and then randomised to undergo a minimally invasive approach to adrenalectomy (i.e. TLA or PRA) for resection of pathologies of the adrenal gland.
- Comparison — Any patient who are eligible and randomised to undergo any of the other approaches (including other minimally invasive/laparoscopic approaches) to adrenalectomy for resection of the same pathologies of the adrenal gland.
- Outcomes

- The primary outcomes of interest were:

- Rates of specific complications experienced post-operatively including surgical site infections (SSIs), pneumothoraces, and haematoma.
- Timing/duration of operation, defined as the time taken in minutes from opening skin to skin closure.
- Intra-operative blood loss, reported in millilitres.
- Rates of conversion to open adrenalectomy following initial attempt to perform the other approaches to adrenalectomy.

- The secondary outcomes of interest were:

- Post-operative pain reported using visual analogue scales (VAS) at 12 h post-operatively (D0), 24 h post-operatively (D2), and 48 h post-operatively (D2).
- Cost of each approach to adrenalectomy (in euros). In cases where cost was provided in another currency, local conversion rates were applied for comparability.
- LOS post-operatively reported in days.
- Recurrence following adrenalectomy — this included oncological, biochemical, or physiological recurrences

### Search strategy

A formal systematic search was performed on the 23rd of December 2021 of the PUBMED, EMBASE, and Cochrane Central Register of Controlled Trials (CENTRAL) electronic databases. Each database was initially searched for relevant titles. This search was performed by two independent reviewers, using a predetermined search strategy that was designed by the senior authors. This search included the following search term: (Adrenalectomy[MeSH Terms]. Boolean operators were not deployed for this search. Manual cross-referencing of reference lists from previous systematic reviews, meta-analyses, and included trials was undertaken. As described, studies were limited to those published in the period 2001–2021.

Manual removal of duplicate studies was performed before all titles were screened. Thereafter, RCTs considered to be appropriate had their abstracts and/or full text reviewed. Retrieved studies were reviewed to ensure inclusion criteria were met for the primary outcome at a minimum, with discordances in opinion resolved through consultation with the third author (NED). Data extraction was also performed by two independent reviewers (MGD & ÉJR), with study details, basic patient clinicopathological characteristics, and surgical data all recorded. The final search was performed in January 2022.

### Data management and analysis

Descriptive statistics were used to outline and report characteristics of included trials. Rates of complications and conversion to open adrenalectomy rates were expressed as dichotomous or binary outcomes, reported as odds ratios (ORs) expressed with 95% confidence intervals (CIs). ORs were calculated, using crude event RCT data, to compare interventions using per-protocol data, where applicable. Comparative operation times, intra-operative blood loss, VAS pain, etc. were calculated using mean values, standard deviations (SD), and pooled mean variance. The mean difference (MD) between approaches to adrenalectomy was then calculated at NMA. The principal comparator varied in several of these analyses based on the approaches to adrenalectomy compared for each primary outcome measure. Cost analyses of each approach to adrenalectomy were performed for the adrenalectomy operation and not for entire length of hospital stay. Analyses were performed to facilitate US Dollars (USD) conversion to euros at a rate of 1.00 USD = 0.88 euro [35] and one-way analysis of variance analyses (or ANOVA, **†**) were performed to compare mean costs of each procedure. All surgical costs are reported in euro. Disease recurrence was defined as ‘invasive disease recurrence of the primary adrenal malignancy for which the patient underwent the surgical resection with adrenalectomy (which subsequently was evaluated in an RCT)’.

Bayesian network meta-analyses were conducted using Netameta and Shiny packages for R [23]. Effect sizes were described with a 95% CI. Results were considered statistically significant at the *P* < 0.050 level if the 95% CI did not include the value of one. Estimates of mean and SDs were calculated using standard statistical methods, where applicable [36, 37]. Rank probabilities were plotted against the possible ranks for all competing treatments. The deviance from unrelated mean effect inconsistency models was used to establish the inconsistency of each analysis performed. Rankings were illustrated using Litmus rank-o-gram surface under the cumulative ranking curve (SUCRA) analyses, with directionality imposed upon outcome measures by author to provide clinical relevance to results. The confidence in estimates of the outcome was assessed using ‘Confidence in Network Meta-Analysis’ (CINeMA) [38]. Methodological assessment of included studies was undertaken using the Cochrane Risk of Bias assessment tool [39]. Quality assessment of included studies was conducted assessed using the GRADE (Grading of Recommendations, Assessment, Development and Evaluations) assessment [40].

## Results

### Literature search and study characteristics

**Fig. 1:**
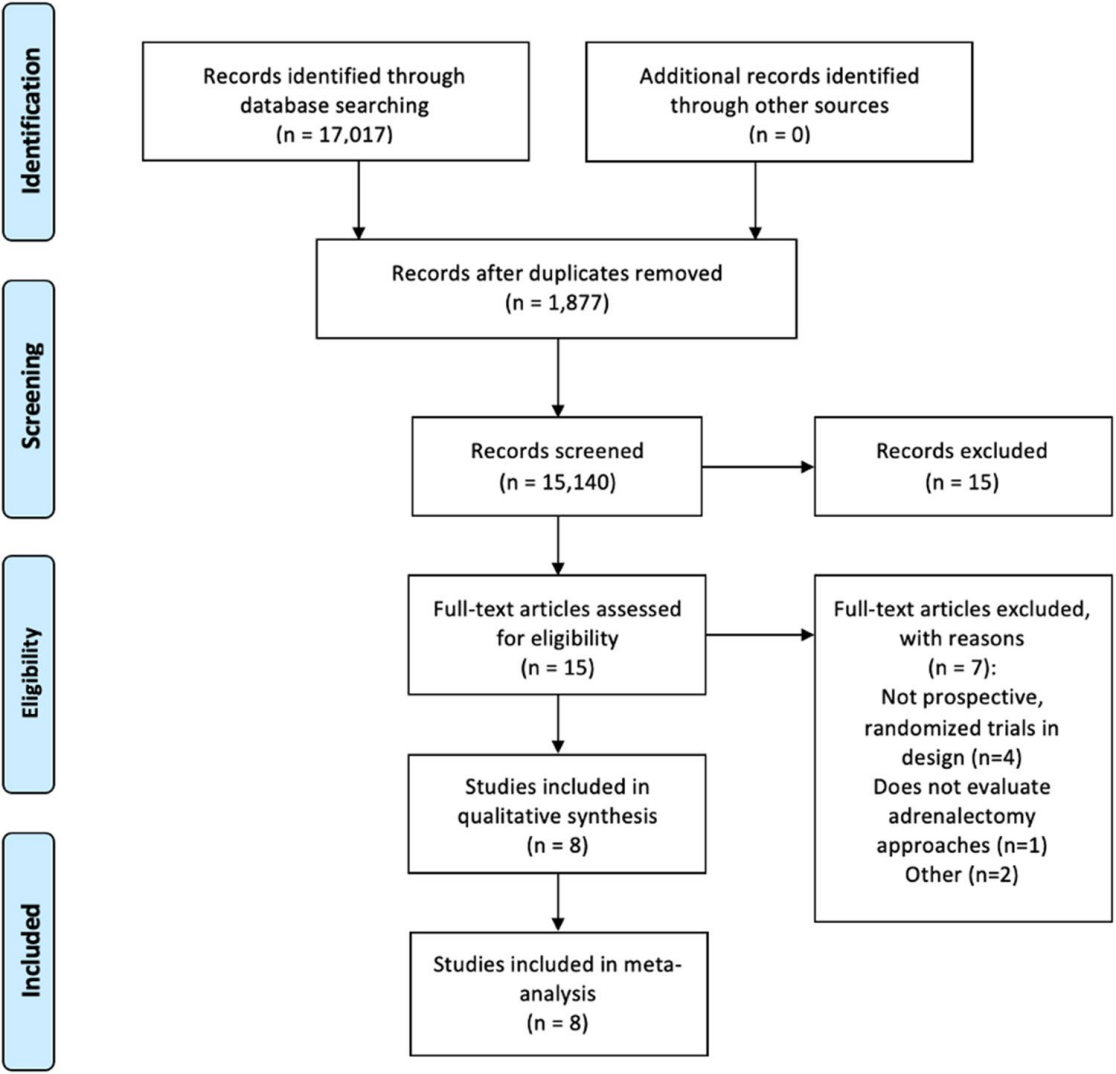
PRISMA flowchart outlining the systematic search process

The systematic search strategy identified a total of 17,017 studies, of which 1877 duplicate studies were manually removed. The remaining 15,140 studies had their titles and/or abstracts screened for relevance, before 15 full texts were reviewed. In total, 8 RCTs fulfilled our inclusion criteria and were included in this systematic review and network meta-analysis [41–48] (Fig. 1). Publication dates ranged from 2004 to 2019 (Table 1). The mean follow up was 33.6 months.

**Table 1:** Table of the eight included randomised controlled trials

Author	Year	Study	Country	*N*	OA	TLA	PRA	RA
Barczynski	2014	RCT	Poland	65	**-**	33	32	**-**
Chai	2017	RCT	Korea	83	**-**	42	41	**-**
Kozlowski	2019	RCT	Poland	77	**-**	33	44	**-**
Ma	2005	RCT	China	140	**-**	50	20	70
Mohammedi-Fallah	2013	RCT	Iran	24	**-**	11	13	**-**
Morino	2004	RCT	Italy	20	**-**	10	**-**	10
Rubinstein	2005	RCT	USA	57	**-**	25	32	**-**
Tiberio	2008	RCT	Italy	22	9	13	**-**	**-**
Total	-	-	-	488	9	217	182	80

*N*, number; *OA*, open adrenalectomy; *TLA*, transabdominal lateral adrenalectomy; *PRA*, posterior retroperitoneal adrenalectomy; *RA*, robotic adrenalectomy; *RCT*, randomised controlled trial

### Clinicopathological and surgical data

In total, there was data included from 488 patients with mean age at diagnosis of 48.9 years (range 19–84 years — 8 studies) [41–48]. Overall, 55.9% of patients were female (273/488 — 8 studies) [41–48]. The mean body mass index of included patients was 26.1 (range: 19–35 — 7 studies) [41–47]. In total, 46.1% of patients underwent resection of phaeochromocytomas (225/488), 26.2% underwent resection of a non-functioning adrenal tumour (128/488), 16.8% underwent resection of an aldosteronoma (82/488), 9.6% underwent resection of a glucocorticoid secreting adrenal adenoma (47/488), and just 6 patients underwent resection of an isolated metastatic deposit or other tumours (including 3 cases of ACC) (1.2%).

Overall, 44.5% of patients underwent TLA (217/488), 37.3% underwent PRA (182/488), 16.4% underwent RA (80/488), and 1.8% patients underwent OA (9/488) (Table 1). More patients underwent left sided adrenalectomy than right sided adrenalectomy (left: 59.8%, 262/458 vs. right: 40.2%, 196/458 — 8 RCTs) [41–48].

The mean size of resected tumours was 35 mm in largest diameter (range: 8–65 mm — 8 studies) [41–48], which was significantly greater for RA compared to laparoscopic approaches (i.e. TLA and PRA): The mean tumour size resected was 44.3 mm for RA, 40.9 mm for OA, 35.5 mm for TLA, and 34.4 mm for PRA (*P* < 0.001, **†**). Clinical data and indications for adrenalectomy from the 8 included RCTs are provided in detail in Table 2.

**Table 2:** Clinical data from the 8 included randomised controlled trials

Author	Year	Female	Male	Age in years (range)	BMI (range)	Right sided	Left sided	Size in mm (range)	Follow-up in months	Indications for surgery	RoB
Barczynski	2014	48	17	47 (41–52)	27 (26–29)	31	34	40 (33–46)	31	Unilateral benign adrenal tumours up to 7 cm in size	Low
Chai	2017	54	29	47	24	36	47	30	31	Unilateral benign adrenal tumours up to 7 cm in size (or less than 5 cm in cases of phaeochromocytoma)	Some
Kozlowski	2019	43	34	60 (32–84)	30	29	48	40 (8–75)	28	Unilateral benign adrenal tumours up to 8 cm in size	Some
Ma	2005	64	76	47 (35–50)	22 (20–25)	38	72	43 (30–60)	46	Unilateral adrenal-originated phaeochromocytoma	Some
Mohammedi-Fallah	2013	14	10	42 (20–57)	27 (22–35)	14	10	27 (20–50)	9	Unilateral benign adrenal tumours up to 6 cm in size	High
Morino	2004	11	9	40 (19–72)	24 (19–31)	14	6	32 (14–65)	**-**	Unilateral benign adrenal tumours up to 10 cm in size	High
Rubinstein	2005	31	26	57 (46–66)	29 (25–33)	21	36	27 (16–49)	72	Unilateral adrenal tumours	Low
Tiberio	2008	8	14	51 (34–74)	**-**	13	9	41 (22–60)	18	Unilateral benign adrenal-originated phaeochromocytoma	Some
		273	215	48.9 (19–84)	26.1 (19–35)	196	262	35 (8–65)	34		-

*BMI*, body mass index; *RoB*, Cochrane risk of bias assessment for prospective randomised studies

### Duration of operation

All 8 RCTs reported on the operative duration of adrenalectomy [41–48]. The shortest operation mean duration for adrenalectomy was for PRA (93.7 min, range: 30–180 min). Conversely, the longest operation duration was OA with a mean of 180.0 min (range: 120–230 min) (Supplementary Material S1). At network meta-analysis, there was no significant difference in operative time reported for OA (MD: − 21.50 (95% CI: − 22.60–66.10)) or PRA (MD: 7.82 (95% CI: − 21.20–36.70)) compared to TLA (2 RCTs) (Supplementary Material 2A). The associated ranking tables for these approaches to adrenalectomy and operative duration are outlined in Table 3A.

**Table 3:** Ranking tables with respect to (A) intra-operative duration, (B) intra-operative blood loss, (C) post-operative complications, (D) conversion to open adrenalectomy, (E) day 0 post-operative pain (measured using visual analogue scoring), and (F) length of hospital stay

(A)	Intra-operative duration			SUCRA
	Rank 1	Rank 2	Rank 3	%
TLA	0.644025	0.3138	0.042175	78%
RPA	0.2115125	0.5238375	0.26465	48%
RA	0.1444625	0.1623625	0.693175	23%
(B)	Intra-operative blood loss			SUCRA
	Rank 1	Rank 2	Rank 3	%
Open	0.7485625	0.22615	0.0252875	88%
TLA	0.2352875	0.7510375	0.013675	59%
RPA	0.01615	0.0228125	0.9610375	3%
(C)	Complications			SUCRA
	Rank 1	Rank 2	Rank 3	%
RA	0.7045625	0.185175	0.1102625	78%
RPA	0.1321375	0.329175	0.5386875	40%
TLA	0.1633	0.48565	0.35105	30%
(D)	Conversion to open			SUCRA
	Rank 1	Rank 2	Rank 3	%
RA	0.74045	0.23195	0.0276	82%
RPA	0.1523375	0.5062	0.3414625	42%
TLA	0.1072125	0.26185	0.6309375	24%
(E)	Day 0 post-operative pain			SUCRA
	Rank 1	Rank 2	Rank 3	%
RPA	0.8224375	0.1775625	-	81%
TLA	0.1775625	0.8224375	-	19%
(F)	Length of hospital stay			SUCRA
	Rank 1	Rank 2	Rank 3	%
RPA	0.880125	0.119875	-	88%
TLA	0.119875	0.880125	-	12%

### Intra-operative blood loss

In total, 7 RCTs reported on intra-operative blood loss during adrenalectomy [41–45, 47, 48]. Patients undergoing OA had the largest intra-operative blood loss at surgery (mean: 164 ml), while those undergoing TLA had the smallest intra-operative blood loss (mean: 50.6 ml, range: 25–200mls) (Supplementary Material S1). At network meta-analysis, there was a significant increase in intra-operative blood loss for those undergoing OA (MD: 117.00 (95% CI: 1.41–230.00)) with similar blood loss observed for those undergoing PRA (MD: − 10.50 (95% CI: − 83.40–65.90)) compared to TLA (3 RCTs) (Supplementary Material 2B). The associated ranking tables for these approaches to adrenalectomy and blood loss are outlined in Table 3B.

### Overall complications

The overall complication rate following adrenalectomy was 12.9% (38/294) from the 4 RCTs included which reported complication rates [43, 44, 46, 47]. RA had the highest complication rates (13.8%, 11/80) and TLA had the lowest complication rates (12.4%, 14/113) (Supplementary Material S3). Unfortunately, specific complications reported by the RCTs included in this study were incompatible with independent NMA.

At NMA for overall complications, there was no significant difference reported for RA (OR: 1.130 (95% CI: 0.404–3.000)) or PRA (OR: 0.657 (95% CI: 0.195–2.200)) compared to TLA (4 RCTs) (Supplementary Material 2C). The associated ranking tables for these approaches to adrenalectomy and post-operative complications are outlined in Table 3C.

Of note, post-operative pneumonia (*n* = 8) and requiring blood transfusions (*n* = 6) were the commonest complications reported post-adrenalectomy, with no studies reporting data on incisional hernia. The breakdown of the reported complications is outlined in Supplementary Material S4.

### Conversion to open

In total, 6 RCTs reported on converting to open during adrenalectomy [41, 43–47]. The overall conversion rate was 2.3% (9/383). RA had the highest conversion to open rates (5.0%, 4/80), compared to PRA (2.1%, 3/146) and TLA (1.3%, 2/157) (Supplementary Material S3). At network meta-analysis, there was no significant difference observed for RA (OR: 7.91 (95% CI: 0.167–396.000)) or PRA (OR: 3.92 (95% CI: 0.231–126.000)) compared to TLA (4 RCTs) (Supplementary Material 2D). Indications for converting to open included right adrenal vein haemorrhage [42] and failure to progress during MIS approach [45, 47]. Ranking tables for minimally invasive approaches to adrenalectomy and conversion to open are outlined in Table 3D.

### Post-operative pain

Overall, 4 RCTs reported post-operative VAS pain scores [41–43, 45] and just RCT reported post-operative pain complicating the procedure as a dichotomous outcome (Supplementary Material S4) [43]. TLA was higher than PRA at D0 post-operatively (mean VAS: 4.4 vs. 3.7), D1 post-operatively (mean VAS: 3.0 vs. 2.1), and D2 post-operatively (mean VAS: 2.5 vs. 1.7) (Supplementary Material S5). At network meta-analysis, there was no significant difference in pain at D0 post-operatively reported for PRA (MD: − 0.423 (95% CI: − 1.350–0.580)) compared to TLA (2 RCTs) (Supplementary Material 2E). Ranking tables for these approaches to adrenalectomy and post-operative pain are outlined in Table 3E.

### Length of hospital stay

**Fig. 2:**
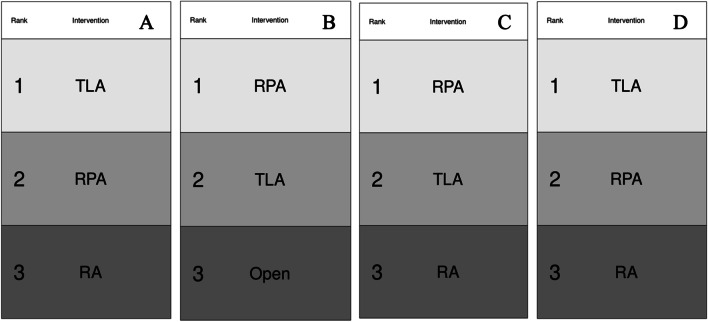
Ranking figures with respect to **A** intra-operative duration, **B** intra-operative blood loss, **C** post-operative complications, and **D** conversion to open adrenalectomy

Overall, 7 RCTs reported LOS following adrenalectomy [41–46, 48]. OA had the longest LOS (mean: 8.0 days), compared to RA (mean: 4.4 days), TLA (mean: 3.6 days), and PRA (mean: 2.1 days) (Supplementary Material S5). At network meta-analysis, there was no significant reduction observed for LOS following PRA (MD: − 0.178 (95% CI: − 0.476–0.149)) compared to TLA (2 RCTs) (Supplementary Material 2F). Ranking tables for these approaches to adrenalectomy and length of hospital stay are outlined in Table 3F. Ranking figures illustrating the most favourable approach for relevant outcome measures are illustrated in Fig. 2. Network plots illustrating the number of RCTs included for the meta-analysis performed for the previous 6 outcome measures are outlined in Supplementary Material S6.

### Disease recurrence

In total, 4 RCTs reported disease recurrence following adrenalectomy [42, 43, 47, 48] and there was just 1 recurrence observed in the patients included (0.4%, 1/239). This recurrence occurred in patient undergoing PRA in the trial performed by Rubenstein et al. (0.8%, 1/117) [47]. Details surrounding this recurrence are not provided in this study (i.e. oncological, biochemical, or physiological recurrence). No patients who underwent OA (0.0%, 0/9) or TLA (0.0%, 0/113) suffered disease recurrence (Supplementary Material S3).

### Cost of adrenalectomy

Overall, 3 RCTs reported cost relating to approach to adrenalectomy [41, 44, 46]. Of these, Ma et al. report cost for full hospitalisation for adrenalectomy [44], which was not included in our analysis. Morino et al. and Barczynski et al. report costs of performing an adrenalectomy and were included in this analysis [41, 46]. The overall mean cost of performing an adrenalectomy was 2729.50 euros. Overall, RA was the most expensive approach to adrenalectomy (RA: 3050 euros vs. TLA: 2362 euros vs. PRA: 1728 euros, *P* < 0.001, **†**) (Supplementary Material S5).

### Risk of bias assessment

The majority of included RCTs had low-to-moderate risk of bias: Overall, 2 of the included RCTs had low-risk of bias (25.0%) [41, 47], 4 of the RCTs included some concerns for bias (50.0%) [42–44, 48], while 2 RCTs were considered high-risk of bias (25.0%) [45, 46]. Risk of bias assessment is summarised in Table 1. Quality assessment of included studies was conducted assessed using the GRADE assessments (Supplementary Material S7). Deviance from unrelated mean effect inconsistency models is outlined in Supplementary Material S8. Rankings illustrated using Litmus rank-o-gram surface under the cumulative ranking curve (SUCRA) analyses are depicted in Supplementary Material S9.

## Discussion

This study is the first systematic review and NMA to integrate data from prospective, randomised clinical trials to evaluate post-operative surgical outcomes following different approaches to adrenalectomy for adrenal lesions suitable for a minimally invasive approach. In total, eight RCTs were included and the raw data demonstrated that there were favourable intra-operative and post-operative outcomes reported for those who underwent TLA and PRA approaches compared to those undergoing OA and RA, despite the indications being similar irrespective of approach used. At NMA, most analyses failed to illustrate significant differences between approaches to adrenalectomy, except for the data illustrating increased blood loss following OA relative to other approaches. Importantly, the size of the tumours resected in each of the included studies was similar for those undergoing OA and other approaches, with none of the adrenalectomies be performed for large, locally advanced, or ACC (except for 3 ACCs resected in the study by Rubenstein — 2/25 from the TLA group and 1/32 from the PRA group). In addition, just Ma et al. and Rubenstein et al. compared approaches for resecting both benign and invasive cancers of the adrenal glands, with just 10.5% (6/57) of cases included by Rubenstein et al. being for malignant pathologies, with no differentiation among pathologies being reported by Ma et al. Therefore, the results of this study highlight the significance of adopting minimally invasive techniques for small, non-ACC-related lesions of the adrenal gland where feasible, when surgical resection is indicated.

In this analysis, TLA and PRA exhibited favourable outcomes with respect to intra-operative duration, blood loss, and post-operative complications compared to patients undergoing OA or RA, while demonstrating lower conversion to open rates for these MIS approaches compared to RA. The most informative results are derived from the crude values reported for each approach to adrenalectomy at systematic review, as the majority of these analyses failed to demonstrate statistical significance at meta-analysis. A plausible explanation for these finding is that NMA calculations may be underpowered due to a lack of data availability for certain outcome measures (e.g. all 8 RCTs report intra-operative duration, yet just 2 of these provide data for integration into meta-analysis). While the NMA element to this study may be scrutinised for being performed prematurely, the systematic review component of this study provides insightful ‘real-world’ data in relation to intra- and post-operative surgical outcomes following adrenalectomy.

Unsurprisingly, patients undergoing OA experienced significantly more intra-operative blood loss compared to their counterparts who underwent minimally invasive adrenalectomy. This is a predictable finding, and one that supports the use of MIS techniques when resecting non-malignant adrenal cancer pathologies, as recommended by the Society of Gastrointestinal and Endoscopic Surgeons (SAGES) guidelines [49]. In addition to the data highlighting reduced blood loss associated with minimally invasive adrenalectomy techniques, MIS is perceived to be advantageous as patients are subject to less physiological stress, immunologic burden, faster recovery times, lower complication rates, and less use of healthcare resources [50–52]. Accordingly, this study further emphasises and validates the current paradigm shift towards adopting MIS techniques where clinically appropriate and possible, reserving OA for larger, locally advanced cases and ACCs, where laparoscopic or robotic surgery may prove futile in achieving a complete R0 resection [12, 53]. Notwithstanding, acknowledgement must be made for the likelihood that this indication for OA may confound the results implying that OA has longer operative time and blood loss relative to minimally invasive approaches to the adrenal gland.

Overall, TLA had the lowest intra-operative blood loss (mean: 51 millilitres per operation), conversion to open rates (1.3%, 14/113), and complication rates (12.4%, 14/113) when compared to other approaches to adrenalectomy. TLA is advantageous as it facilitates the surgeon to work with typically familiar intra-bdominal anatomy and in a wider working space than PRA [54], while making use of what the majority of surgeons would consider more familiar instrumentation than robotic instruments [55]. In addition, TLA is associated with reduced complication rates than is observed in patients who have undergone OA [28, 56]. When considering these factors, this RCT data illustrating the benefit of TLA approach may be unsurprising; however, several of these outcomes are comparable for PRA: We observed a minimal difference in intra-operative blood loss for TLA and PRA (mean: 51 ml vs. 55 millilitres per operation), which is unlikely to be clinically relevant and PRA had the shortest intra-operative duration, length of hospital stay, and lowest post-operative pain. As such, when MIS is indicated, PRA may be considered once surgical and institutional expertise allows, as previously demonstrated by Nigri et al. in their meta-analysis [29, 57]. Thus, this study supports the use of minimally invasive laparoscopic/retroperitoneoscopic approaches to adrenalectomy where indicated, with reservation of the OA approach for cases where minimally invasive approaches are deemed be unlikely to effectively resect the tumour to establish oncological control, for example, for large (> 8 cm), locally advanced tumours, those extending into major venous structures, or those of ACC pathology [22, 53].

While there are several potential advantages of adopting the RA approach compared to other minimally invasive approaches (TLA/RPA) [14, 15, 58], the results of the current meta-analysis do not support the adoption of RA as routine at present. There are several reasons for such results: In this analysis, two RCTs compared RA and minimally invasive techniques [44, 46]: Morino et al. conducted the first RCT comparing RA and MIS techniques, which provided very disappointing results, with 40.0% of the RA group requiring conversion to open surgery to successfully resect the adrenal gland [46]. Subsequently, Ma et al. performed a second RCT with more promising results regarding the use of RA (less patients converted to open (0/70 vs. 1/70 for LA) and reduced mean intra-operative time (92.5 min vs. 122.5 min for LA) [44], which has been supported by several recent observational studies [59, 60]. Furthermore, there were only 80 patients who underwent RA in this analysis which may bring into question the robustness of these results. At the time of writing, these early RCTs are approaching 20 years since publication, and it is likely that robotic techniques have become optimised as the learning curve is negated in recent years. Therefore, once this steep learning curve effect associated with RA is overcome [61], the timing may be appropriate to conduct large, prospective multicentre RCTs which may prove insightful as to whether RA or the other minimally invasive approaches (TLA/PRA) best serve patients by improving surgical outcomes following adrenalectomy. In particular, surgeons would be likely have to developed a certain degree of familiarity with the static camera vision, motion scaling, and tremor elimination offered by robotic surgery [62], while allowing time for the reported disadvantages such as increased costs, longer operative times, and loss of haptic feedback [63, 64] to be offset as the learning curve is surmounted [65].

As previously described, there are no previous reports of prospective, randomised trials comparing the safety of all four approaches to adrenalectomy. Thus, the authors deployed NMA methodology to evaluate the previously published RCTs to establish the surgical and oncological safety profiles of each of approach to adrenalectomy. Heger et al. previously performed a NMA of both randomised and non-randomised clinical trials evaluating outcomes following open and MIS approaches to adrenalectomy [29]. Results from the current study are congruent with the work of Heger et al., illustrating the advantages of MIS in reducing intra-operative blood loss, post-operative complications, and LOS following adrenalectomy. Conversely, Heger et al. report OA as the fastest technique, directly contradicting the results of the current analysis. It is of interest to note that there have been several previous meta-analyses demonstrating comparable outcomes for RA and other minimally invasive approaches: Brandao et al. performed a meta-analysis of observational studies including 600 patients which demonstrated comparable surgical outcomes for RA and minimally invasive adrenalectomy [27]. Similarly, Economopoulos et al. illustrated the safety of RA relative to laparoscopic adrenalectomy in their extensive meta-analysis performed involving 1162 patients from 27 studies [26]. As techniques in robotic endocrine surgery become more commonly used, these previous meta-analyses support robust adoption of RA where feasible, yet the current study successfully challenges this work from these previous authors using RCT data. As previously outlined, the modest outcomes observed in our study in relation to RA suggest there remains a steep learning curve for RA adoption as ‘gold standard’ [61]. Additionally, there are several meta-analyses which compare surgical outcomes for OA and minimally invasive techniques [13, 28, 30, 66]: Sgourakis et al. previously reported similar surgical and post-operative outcomes for those undergoing OA and MIS for stage I/II adrenocortical carcinoma, with poorer 5-year survival outcomes observed for those undergoing minimally invasive techniques [30]. In their recent meta-analysis of 14 studies and 743 patients, Li et al. outlined minimally invasive adrenalectomy as a more feasible and safer treatment option to OA, with reduced post-operative morbidity and improved recovery outcomes observed following MIS in those undergoing resection of phaeochromocytoma [28]. Similarly, Fu et al. confirmed the efficacy of MIS for phaeochromocytoma surgery in their recent meta-analysis [13]. While this study focuses on studies of the highest level of evidence to definitively establish the optimal approach to adrenalectomy, Perivoliotis et al. performed a previous analysis of 2997 patients (from 21 studies of moderate-to-high quality evidence — 1 RCT and 20 non-randomised studies) which ultimately favoured RA as the optimal approach to adrenalectomy [66].

### Limitations

Despite several strengths, the current study is subject to several inherent limitations. Firstly, as previously outlined, several outcome measures used in our NMA may be underpowered relative to the robust availability of crude data reported at systematic review, limiting the robustness of NMA results. Secondly, the year of publication of RCTs was intentionally limited to those published in the past 20 years (2001–2021) to ensure the modern adrenal surgery paradigm was within the data used for this analysis. Although this was performed pragmatically by the authors, it is possible remains that some RCTs may have not been captured in this search. Thirdly, with just 9 patients undergoing OA in the current analysis, it is obvious that this analysis is not powered to provide many meaningful comparisons between OA and other approaches to adrenalectomy. Therefore, caution must be taken when interpreting results described in relation to this method of adrenalectomy. Finally, in the wake of the era of robotic endocrine surgery, we must re-emphasise the steep learning curve associated with RA. As robotic surgical techniques become optimised, future RCTs will likely provide more insightful and exciting results in relation to robotic approaches to the adrenal gland, as we strive towards an era of minimally invasive, personalised oncological care.

In conclusion, the current systematic review and NMA of RCTs highlights the value of adopting laparoscopic approaches in achieving favourable surgical and oncological outcomes following adrenalectomy. While outcomes post-OA are inferior relative to minimally invasive adrenalectomy, judicious case selection is warranted to determine which cases would benefit from open approaches in the era of minimally invasive surgery. While the results of this study fail to support RA as routine, the learning curve associated with RA may have impacted the results and this approach is likely to play a role in future MIS of the adrenal gland. Therefore, contemporary RA warrants robust investigation in well-designed designed RCTs to determine its position in the multimodal management paradigm of pathologies of the adrenal gland.

### Supplementary information

Below is the link to the electronic supplementary material.

Supplementary file1 (DOCX 681 KB)
