# Supplementary material for: Comparing surgical outcomes of approaches to adrenalectomy — a systematic review and network meta-analysis of randomised clinical trials
Source: Langenbecks Arch Surg. 2023 May 5;408(1):180. doi: 10.1007/s00423-023-02911-7 (PMC10163131; doi:10.1007/s00423-023-02911-7)
Supplement: Supplementary file 1 — Supplementary file1 (DOCX 681 KB) [file 423_2023_2911_MOESM1_ESM.docx]

**Comparing Surgical Outcomes of Approaches to Adrenalectomy – A Systematic Review and Network Meta-Analysis of Randomized Clinical Trials**

Matthew G. Davey PhD MRCSI^1^, Éanna J. Ryan MD MRCSI^2^, Noel E. Donlon MRCSI PhD^2^, Odhrán K. Ryan MB BCh^3^, Mohammed Al Azzawi MCh MRCSI ^2^, Michael R. Boland MCh FRCSI^2^, Michael J. Kerin MCh, FCRSI^1^, Aoife J. Lowery PhD FRCSI^1^

^1^Discipline of Surgery, The Lambe Institute for Translational Research, National University of Ireland, Galway, Galway, Ireland H91YR71

^2^Royal College of Surgeons in Ireland, 123 St. Stephens Green, Dublin 2, Ireland D02YN77

^3^Surgical Professorial Unit, St. Vincent’s University Hospital, Elm Park, Dublin 4, Ireland D04 T6F4

**Supplementary Material**

**Supplementary Material S1.**

Intraoperative data from the 8 included randomized clinical trials.

| Author | Year | Intraoperative Duration in Minutes (Range) | | | | Intraoperative Blood Loss in Millilitres (Range) | | | |
| --- | --- | --- | --- | --- | --- | --- | --- | --- | --- |
|  |  | OA | TLA | PRA | RA | OA | TLA | PRA | RA |
| Barczynski | 2014 | - | 77 (71-84) | 51 (45-57) | - | - | 53 (34-71) | 98 (77-118) | - |
| Chai | 2017 | - | 60 (SD: 18.6) | 68 (SD: 28.7) | - | - | 16 (SD: 25.4) | 11 (SD: 22.1) | - |
| Kozlowski | 2019 | - | 70 (30-140) | 65 (30-140) | - | - | 21(SD: 15) | 7 (SD:5) | - |
| Ma | 2005 | - | 123 (85-165) | 123 (85-165) | 93(75-130) | - | 100 (50-200) | 100 (50-200) | 100 (50-113) |
| Mohammedi-Fallah | 2013 | - | 129 (100-180) | 128 (100-150) | - | - | 66 (50-150) | 66 (50-150 | - |
| Morino | 2004 | - | 115 | - | 169 | - | - | - | - |
| Rubinstein | 2005 | - | 130 (11-187) | 127(90-180) | - | - | 50 (25-100) | 50 (25-100) | - |
| Tiberio | 2008 | 180 (120-230) | 158 (70-270) | - | - | 164 (SD:94) | 48 (SD: 36) | - | - |
|  |  | 180 (120-230) | 107.8 (11-270) | 93.7 (30-180) | 131(75-130) | 164 | 50.6 (25-200) | 55.3 (25-200) | 100 (50-113) |

OA; open adrenalectomy, TLA; transabdominal lateral adrenalectomy, PRA; posterior retroperitoneal adrenalectomy, RA; robotic adrenalectomy, SE; standard error

**Supplementary Material S2.**

Network meta-analyses forest plots with respect to (A) intraoperative duration, (B) intraoperative blood loss, (C) postoperative complications, (D) conversion to open adrenalectomy, (E) Day 0 postoperative pain (measured using visual analogue scoring), and (F) length of hospital stay.


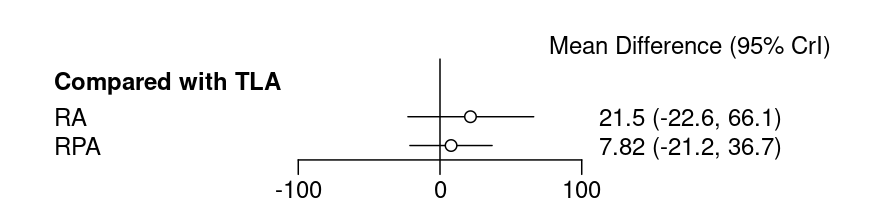

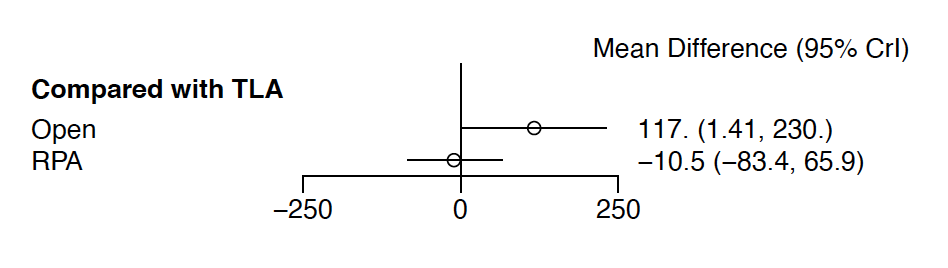


A

B


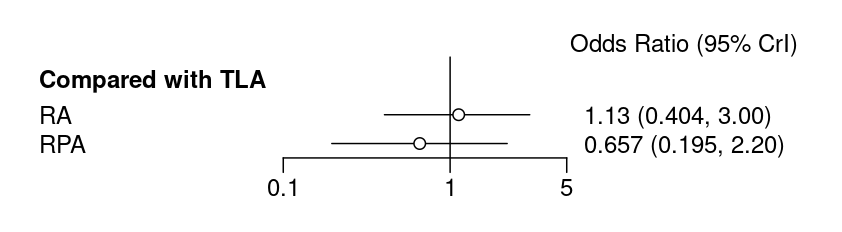

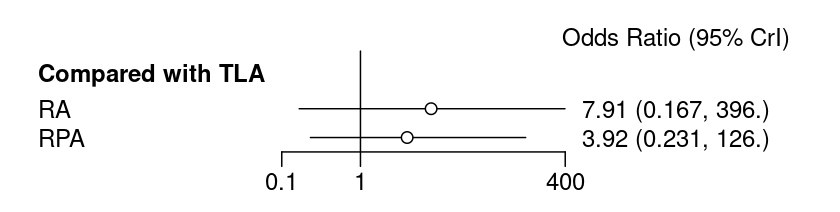


C

D


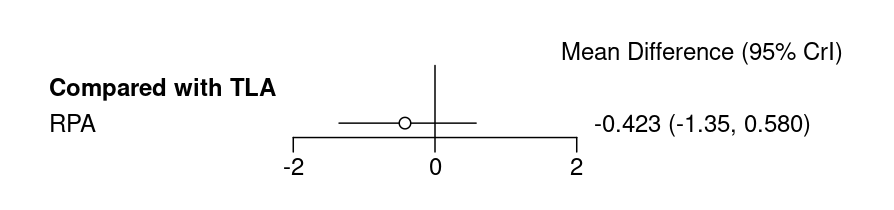

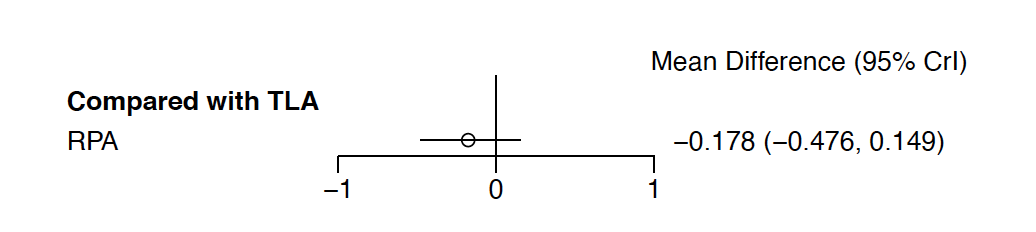


E

F

**Supplementary Material S3.**

Conversion to open and complication rates from the 8 included randomized clinical trials.

| Author | Year | Conversion to Open | | | | Complications | | | | Recurrence | | | |
| --- | --- | --- | --- | --- | --- | --- | --- | --- | --- | --- | --- | --- | --- |
|  |  | OA | TLA | PRA | RA | OA | TLA | PRA | RA | OA | TLA | PRA | RA |
| Barczynski | 2014 | N/A | 0 | 0 | - | - | - | - | - | - | - | - | - |
| Chai | 2017 | N/A | - | - | - | - | - | - | - | - | 0 | 0 | - |
| Kozlowski | 2019 | N/A | 0 | 0 | - | - | 4 | 4 | - | - | 0 | 0 | - |
| Ma | 2005 | N/A | 1 | 1 | 0 | - | 8 | 8 | 11 | - | - | - | - |
| Mohammedi-Fallah | 2013 | N/A | 0 | 1 | - | - | - | - | - | - | - | - | - |
| Morino | 2004 | N/A | 0 | - | 4 | - | 0 | - | 0 | - | - | - | - |
| Rubinstein | 2005 | N/A | 1 | 1 | - | - | 2 | 1 | - |  | 0 | 1 | - |
| Tiberio | 2008 | N/A | - | - | - | - | - | - | - | 0 | 0 | - | - |
|  |  | N/A | 2/157 | 3/146 | 4/80 | - | 14/113 | 13/101 | 11/80 | 0/9 | 0/113 | 1/117 | - |

OA; open adrenalectomy, TLA; transabdominal lateral adrenalectomy, PRA; posterior retroperitoneal adrenalectomy, RA; robotic adrenalectomy, N/A; not applicable

**Supplementary Material S4.**

Breakdown of the complications experienced by patients in this study.

| Author | Pain (longer than 7 days) | Pneumonia | Blood transfusion | Pyrexia | Haematoma | Urinary Retention |
| --- | --- | --- | --- | --- | --- | --- |
| RA | - | 7 | 4 | - | - | - |
| TLA | 4 | - | - | 1 | 1 | - |
| PRA | 4 | 6 | 2 | - | - | 1 |
| OA | - | - | - | - | - | - |

OA; open adrenalectomy, TLA; transabdominal lateral adrenalectomy, PRA; posterior retroperitoneal adrenalectomy, RA; robotic adrenalectomy,

**Supplementary Material S5.**

Postoperative data from the 8 included randomized clinical trials.

| Author | Year | Pain D0 Postoperative (Range) | | | | Length of Hospital Stay in Days (Range) | | | | Cost in Euros | | |  |
| --- | --- | --- | --- | --- | --- | --- | --- | --- | --- | --- | --- | --- | --- |
|  |  | OA | TLA | PRA | RA | OA | TLA | PRA | RA | OA | TLA | PRA | RA |
| Barczynski | 2014 | - | 2.8 (2.5-3.0) | 1.4 (1.2-1.7) | - | - | 4.4 | 2.9 | - | - | 2315 (2192-2436) | 1728 (1622-1834) | - |
| Chai | 2017 | - | 5.1 (SE:1.6) | 5.3 (1.7) | - | - | 2.2 (SE: 0.9) | 2.2 (SE: 0.4) | - | - | - | - | - |
| Kozlowski | 2019 | - | 4.3 (SE: 1) | 3.4 (1) | - | - | 1.4 (SE: 0.5) | 1.1 (SE: 0.4) | - | - | - | - | - |
| Ma | 2005 | - | - | - | - | - | 3 (2-4) | 3 (2-4) | 3 (2-4) | - | - | - | - |
| Mohammedi-Fallah | 2013 | - | 5.5 | 4.8 | - | - | 3.6 (2-5) | 3.1 (2-4) | - | - | - | - | - |
| Morino | 2004 | - | - | - | - | - | 5.4 (4-8) |  | 5.7 (4-9) | - | 2409 | - | 3050 |
| Rubinstein | 2005 | - | - | - | - | - | - | - | - | - | - | - | - |
| Tiberio | 2008 | - | - | - | - | 8 | 5 | - | - | - | - | - | - |
|  |  | - | 4.4 | 3.7 | - | 8 | 3.6 | 2.1 | 4.4 | - | 2362 (2192-2436) | 1728 (1622-1834) | 3050 |

OA; open adrenalectomy, TLA; transabdominal lateral adrenalectomy, PRA; posterior retroperitoneal adrenalectomy, RA; robotic adrenalectomy, SE; standard error

**Supplementary Material S6.**

Ranking figures with respect to (A) intraoperative duration, (B) intraoperative blood loss, (C) postoperative complications, (D) conversion to open adrenalectomy, (E) pain on day 0 postoperatively, and (F) length of hospital stay.

A

C

B

F

E

D

**Supplementary Material S7.**

Quality assessment of included studies and outcome measures conducted using the GRADE (Grading of Recommendations, Assessment, Development and Evaluations) assessment.

| **Outcome** | **Effects and confidence in the estimate of effects** | | | **Comments** |
| --- | --- | --- | --- | --- |
|  | RA | RPA | OA |  |
| **Intraoperative Duration** | | | | |
|  | 21.5 (-22.61, 66.08) Confidence of estimate: ⊕⊕OO Low due to Indirectness | 7.82 (-21.21, 36.73)  Confidence of estimate: ⊕⊕OO Low due to Indirectness | N/A | No difference in risk established |
| Rank | Rank 2/2 | Rank 1/2 | - |  |
| **Estimated Blood Loss** | | | | |
|  | N/A | -10.46 (-83.38, 65.89) Confidence of estimate: ⊕⊕OO Low due to Indirectness | 116.59 (1.41, 229.93)  Confidence of estimate: ⊕OOO Very low due to Indirectness | Significant difference in estimated blood loss for those undergoing OA |
| Rank | - | Rank 2/2 | Rank 1/2 |  |
| **Complications** | | | | |
|  | 1.13 (0.4, 3)  Confidence of estimate: ⊕⊕OO Low due to Indirectness | 0.66 (0.2, 2.2)  Confidence of estimate: ⊕⊕OO Low due to Indirectness | N/A | No difference in risk established |
| Rank | Rank 2/2 | Rank 1/2 | N/A |  |
| **Conversion to Open** | | | | |
|  | N/A | 4.09 (0.24, 116.62) Confidence of estimate: ⊕OOO Very low due to Indirectness | 8.28 (0.18, 400.21) Confidence of estimate: ⊕OOO Very low due to Indirectness | No difference in risk established |
| Rank | N/A | Rank 2/2 | Rank 1/2 |  |
| **Pain on day 0 Postoperatively** | | | | |
|  | N/A | -0.42 (-1.35, 0.58)  Confidence of estimate: ⊕⊕OO Low due to Indirectness | N/A | Unable to analyse |
| Rank | N/A | Rank 1/1 | N/A |  |
| **Length of Hospital Stay** | | | | |
|  | N/A | -0.18 (-0.48, 0.15)  Confidence of estimate: ⊕⊕OO Low due to Indirectness | N/A | Unable to analyse |
| Rank | N/A | Rank 3/4 | N/A |  |

OA; open adrenalectomy, TLA; transabdominal lateral adrenalectomy, PRA; posterior retroperitoneal adrenalectomy, RA; robotic adrenalectomy, N/A; not applicable

**High** = This research provides a very good indication of the likely effect. The likelihood that the effect will be substantially different^‡^ is low.

**Moderate** = This research provides a good indication of the likely effect. The likelihood that the effect will be substantially different^‡^ is moderate.

**Low** = This research provides some indication of the likely effect. However, the likelihood that it will be substantially different^‡^ is high.

**Very low** = This research does not provide a reliable indication of the likely effect. The likelihood that the effect will be substantially different^‡^ is very high.

^‡^ Substantially different = a large enough difference that it might affect a decision.

**Supplementary Material S8.**

Deviance from unrelated mean effect inconsistency models for (A) intraoperative duration, (B) intraoperative blood loss, (C) postoperative complications, (D) conversion to open adrenalectomy, (E) pain on day 0 postoperatively, and (F) length of hospital stay.


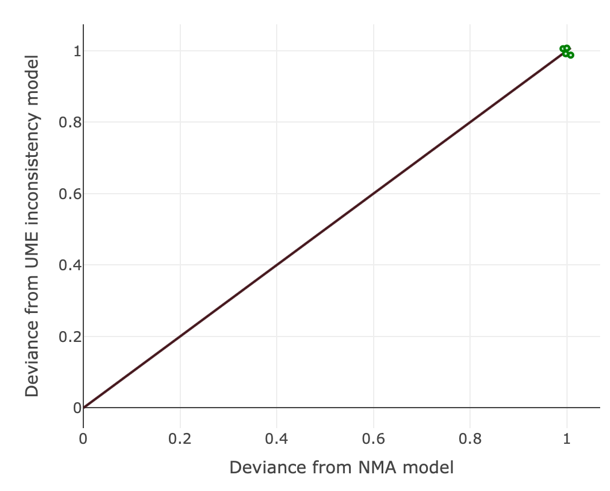

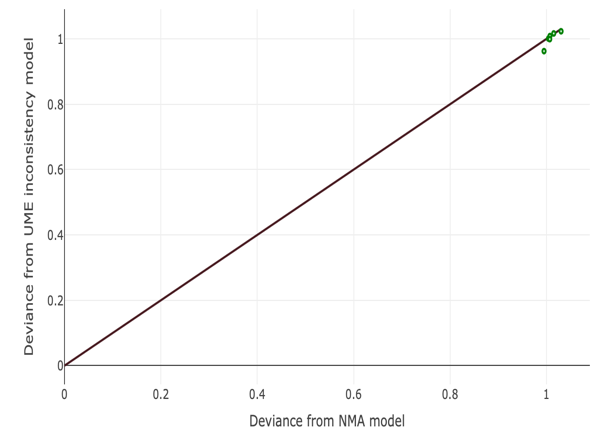

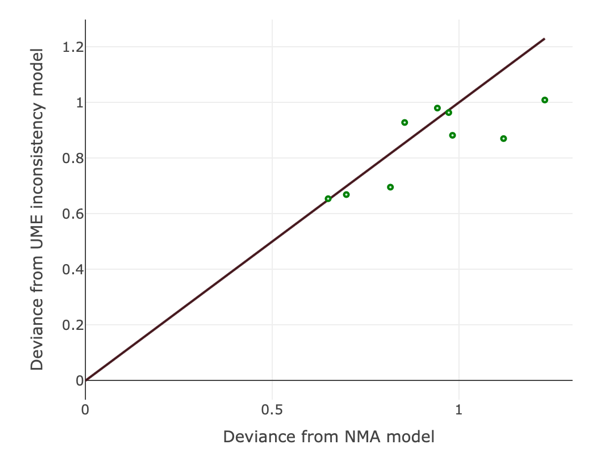


C

B

A


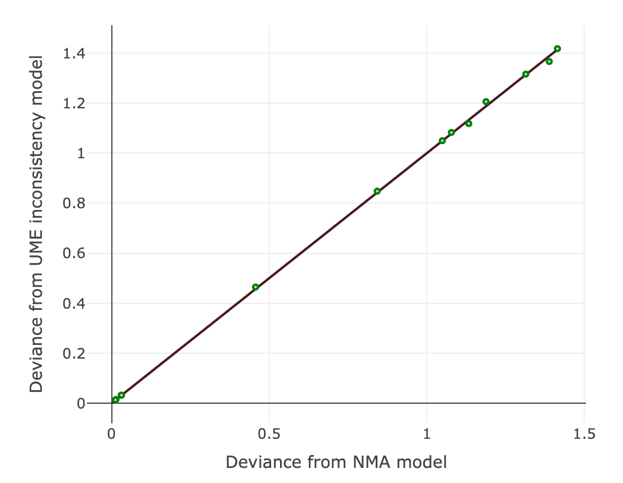

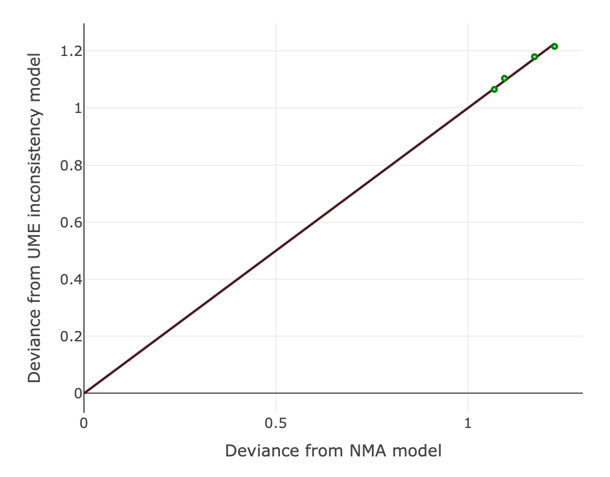

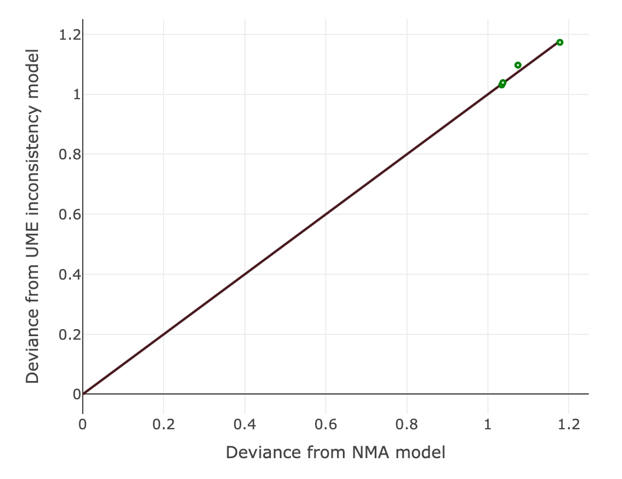


F

E

D

**Supplementary Material S9**.

Rankings illustrated using Litmus rank-o-gram surface under the cumulative ranking curve (SUCRA) analyses for (A) intraoperative duration, (B) intraoperative blood loss, (C) postoperative complications, (D) conversion to open adrenalectomy, (E) pain on day 0 postoperatively, and (F) length of hospital stay.


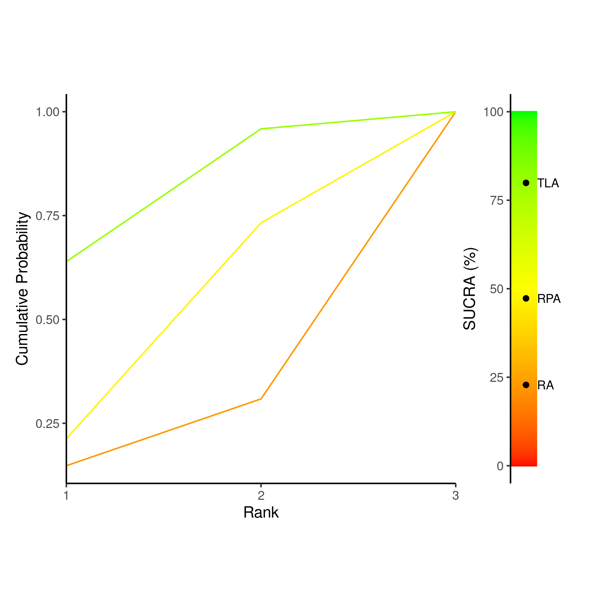

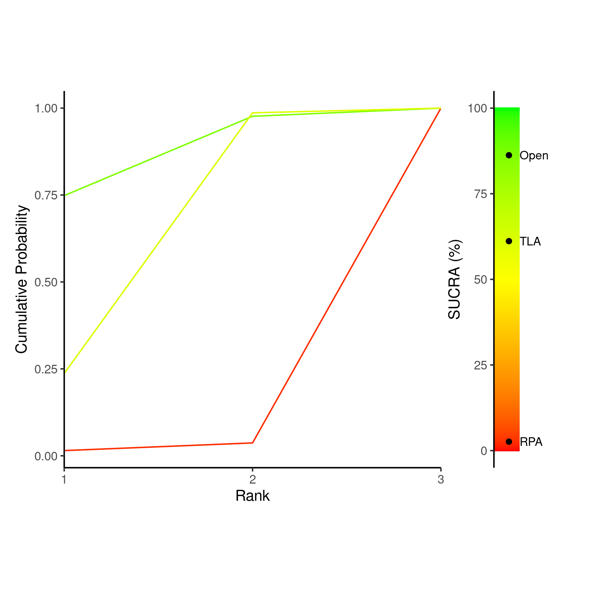

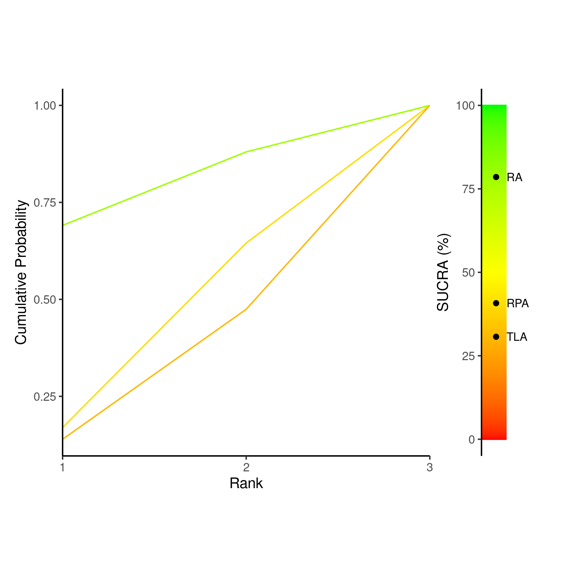

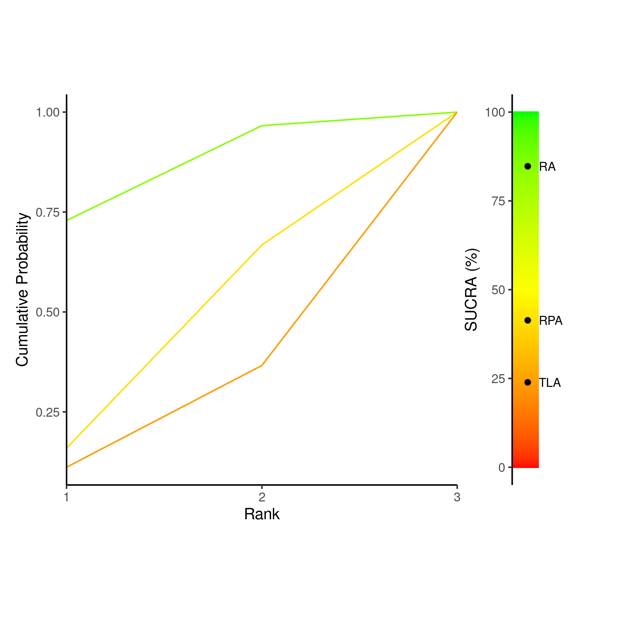

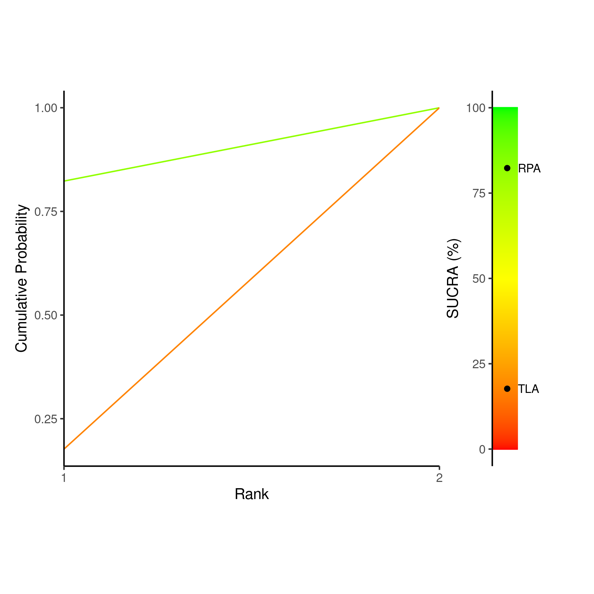

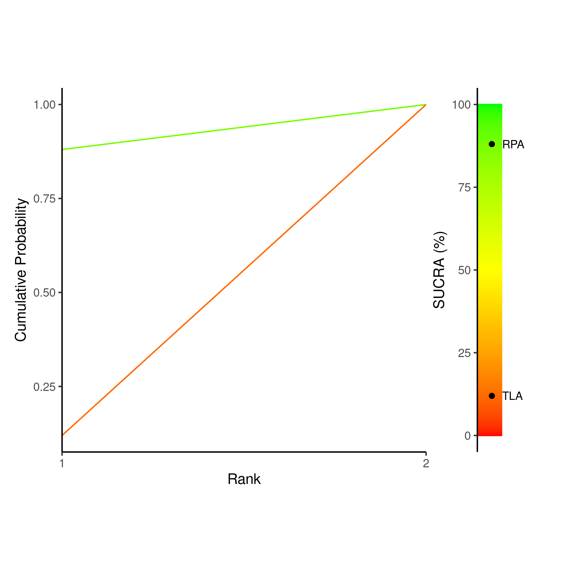


F

E

D

C

B

A
